# Corticospinal gating during action preparation and movement in the primate motor cortex

**DOI:** 10.1152/jn.00639.2017

**Published:** 2018-01-03

**Authors:** Demetris S. Soteropoulos

**Affiliations:** Institute of Neuroscience, Newcastle University Medical School, Newcastle upon Tyne, United Kingdom

**Keywords:** corticospinal system, gating, preparatory delay

## Abstract

During everyday actions there is a need to be able to withhold movements until the most appropriate time. This motor inhibition is likely to rely on multiple cortical and subcortical areas, but the primary motor cortex (M1) is a critical component of this process. However, the mechanisms behind this inhibition are unclear, particularly the role of the corticospinal system, which is most often associated with driving muscles and movement. To address this, recordings were made from identified corticospinal (PTN, *n* = 94) and corticomotoneuronal (CM, *n* = 16) cells from M1 during an instructed delay reach-to-grasp task. The task involved the animals withholding action for ~2 s until a GO cue, after which they were allowed to reach and perform the task for a food reward. Analysis of the firing of cells in M1 during the delay period revealed that, as a population, non-CM PTNs showed significant suppression in their activity during the cue and instructed delay periods, while CM cells instead showed a facilitation during the preparatory delay. Analysis of cell activity during movement also revealed that a substantial minority of PTNs (27%) showed suppressed activity during movement, a response pattern more suited to cells involved in withholding rather than driving movement. These results demonstrate the potential contributions of the M1 corticospinal system to withholding of actions and highlight that suppression of activity in M1 during movement preparation is not evenly distributed across different neural populations.

**NEW & NOTEWORTHY** Recordings were made from identified corticospinal (PTN) and corticomotoneuronal (CM) cells during an instructed delay task. Activity of PTNs as a population was suppressed during the delay, in contrast to CM cells, which were facilitated. A minority of PTNs showed a rate profile that might be expected from inhibitory cells and could suggest that they play an active role in action suppression, most likely through downstream inhibitory circuits.

## INTRODUCTION

The act of suppressing or withholding an upcoming movement until a given time point, also known as motor inhibition, is a critical component of voluntary motor control. A daily example is being able to withhold pressing the gas pedal until the traffic light goes green.

Although there may be no overt movements during a delay period, in terms of brain activity this is not a passive process. Recordings of neural activity in monkeys during instructed delay tasks reliably show that cortical sensorimotor areas including, but not limited to, the primary motor cortex (M1) (Riehle et al. 1985; Riehle and Requin 1989; Tanji and Evarts 1976), the premotor cortex (Churchland et al. 2006b; Crammond and Kalaska 2000; Kalaska and Crammond 1995; Kurata and Wise 1988; Messier and Kalaska 2000; Weinrich and Wise 1982; Wise and Kurata 1989), and the supplementary motor area (Chen et al. 2010; Crutcher et al. 2004; Hoshi and Tanji 2004; Russo et al. 2002; Scangos et al. 2013; Stuphorn and Schall 2002) are highly active during the delay period. This preparatory activity can be correlated with various parameters of the ensuing movement (Churchland et al. 2006a, 2006b; Crammond and Kalaska 2000; Kalaska and Crammond 1995; Riehle and Requin 1989), but that is not always the case (Shen and Alexander 1997a, 1997b). Furthermore, the relationship between the activity seen during preparatory delays and during movement is not straightforward—the activity of a neuron during the delay period does not necessarily predict its activity during movement (Churchland et al. 2006b; Crammond and Kalaska 2000). And yet, M1 is critical for movement execution. Neural activity in M1 is strongly correlated during movement with various kinematic movement parameters (Fagg et al. 2009; Flament and Hore 1988; Holdefer and Miller 2002; Kalaska et al. 1989; Reina et al. 2001; Townsend et al. 2006, and it also has the densest projections to the spinal cord (Coulter and Jones 1977), in addition to potent connections directly onto motoneurons (Lawrence et al. 1985; Muir and Porter 1976; Porter 1985). Damage to M1 or the corticospinal system can produce profound deficits in motor control and permanent muscle weakness, particularly for distal muscles (Lang and Schieber 2004; Lawrence and Kuypers 1968).

This has led to the question, how can M1 neurons, being an integral part of the neural machinery that brings about movement, show delay-related activity without any movement taking place? An obvious explanation is that some form of gating prevents neural activity during movement preparation from being translated into action. The precise nature of this gating, though, is far from clear and is the subject of debate, as multiple possibilities exist. One suggestion is through the presence of an inhibitory mechanism (Cisek 2006; Pouget et al. 2017; Prut and Fetz 1999; Sinclair and Hammond 2009) whereby during a preparatory delay there is an active process of inhibition operating in (but not restricted to) M1 that prevents neural activity from translating into action (“inhibitory gating”). An alternative suggestion stems from the view of the motor neural circuitry as a dynamical system (Churchland et al. 2010; Fetz 1992; Scott 2008; Todorov and Jordan 2002). Within this framework (Kaufman et al. 2014), neural activity can still be modulated at the single-neuron level during the delay period, but as a population these neural responses cancel out at the target structure and do not lead to movement (“dynamical gating”). Both suggestions are appealing. The inhibitory gating mechanism relies on anatomically established neural populations (such as inhibitory interneurons and excitatory projection cells), making relatively clear predictions on how some of these populations should behave during a delay period and during movement. On the other hand, the dynamical systems view allows interpretation of neural firing as a population rather than as single neurons. However, as a relatively new framework within movement neuroscience, it has yet to make clear predictions on what the expected responses of anatomically separate neuronal populations within M1 would be for delay tasks.

Evidence for a gating mechanism during action preparation is readily seen in humans with transcranial magnetic stimulation (TMS). During instructed delay paradigms, suppression of muscle responses to TMS can be reliably observed up until just before the GO signal (Federico and Perez 2017; Greenhouse et al. 2015b; Hasbroucq et al. 1997; Labruna et al. 2014; Lebon et al. 2016; Touge et al. 1998; see Duque et al. 2017 for a comprehensive review and extensive references). As TMS activates the corticospinal system (Burke et al. 1993; Edgley et al. 1990, 1997), the reduction in response amplitude in muscles is taken as a signature for motor inhibition within M1 and provides some support for inhibitory gating. Although evidence for inhibition has been reported in other systems and species (Pouget et al. 2017), it has been much harder to observe in the monkey motor system during similar tasks (Kaufman et al. 2010, 2013)—recordings from neurons in M1 fail to reveal the presence of inhibitory gating, even when attempts have been made to identify putative inhibitory interneurons.

One possible explanation for the discrepancy is that gating is unevenly distributed across all neural subpopulations in M1—human TMS studies selectively probe the corticospinal system, while monkey studies likely sample from a much more diverse population of M1 cells, thus making suppression harder to detect. In addition to corticospinal cells, there are many other pyramidal cell populations such as corticostriatal, corticothalamic, and corticoreticular, and many of these are distinct and nonoverlapping groups of neurons (Otis et al. 2017; Swadlow 1994; Swadlow and Weyand 1981; Turner and DeLong 2000). Current theories regarding action preparation make few, if any, predictions regarding the behavior of these populations—some could show less suppression or even facilitation during action preparation. If so, sampling randomly across all of these groups would possibly result in little evidence for suppression at the population level. Regardless of how these other M1 populations are behaving during a delay, M1 corticospinal outflow, as assessed by TMS in humans, is suppressed during action preparation—thus to look for evidence for suppression in M1, the corticospinal system would be the best place to start.

In this study, the activity of identified M1 corticospinal cells was recorded from two monkeys trained to carry out an instructed delay reach-to-grasp task. The aim was to test for evidence of suppression of corticospinal firing during the task in the output neurons of M1 and, if present, whether this suppression showed any relationship to the onset of the upcoming movement.

## METHODS

All animal procedures were performed under UK Home Office regulations in accordance with the Animals (Scientific Procedures) Act (1986) and were approved by the relevant Local Research Ethics Committee.

### Behavioral Task

**Fig. 1.**
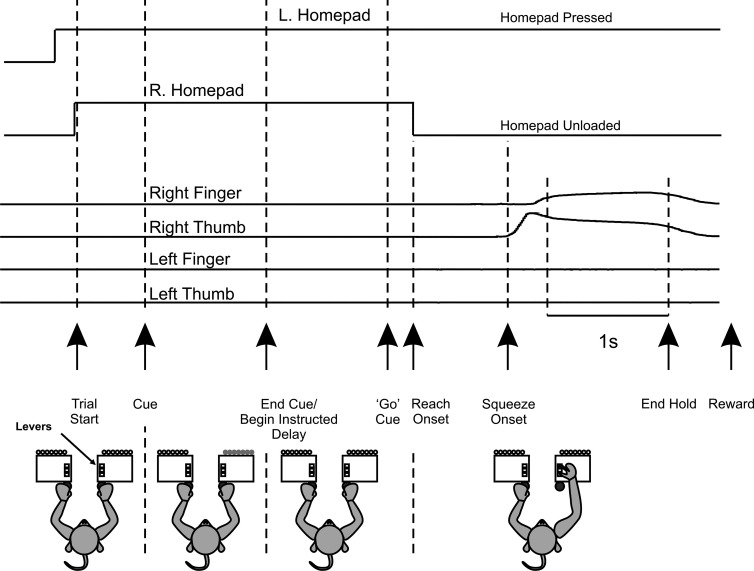
Instructed delay task. Animals initiated a trial by placing both hands on homepads. The state of the homepads is indicated by *top* 2 traces. *Bottom* 4 traces correspond to the position signals for each finger lever. After ~0.5 s from homepad press, there was a 1-s audiovisual laterality cue that indicated to the animal which hand to use. This was followed by an instructed delay period (~1 s), after which time both flags obstructing the levers retracted, allowing the animal to reach and squeeze finger and thumb levers for 1 s, after which they received a food reward.

Two female rhesus macaques (*monkeys T* and *E*; ~4 yr old, ~6 kg) were trained on an instructed delay reach-to-precision grip task (Fig. 1) described previously (Soteropoulos et al. 2011; Soteropoulos and Baker 2006, 2007). The animal was presented with two precision grip manipulanda, one for each hand. Access to the manipulanda was obstructed by plastic flags. The monkey commenced a trial by placing both hands on homepad switches in front of the flags. After ~500 ms, a 1-s-long audiovisual cue indicated the required movement (left hand only, right hand only, or bimanual), chosen at random. After an instructed delay period (0.7–1.3 s), during which the animal had to keep the hands on the homepad switches, both flags then moved down (“GO cue”), permitting access to the manipulanda. The animal had to initiate a reach within 1 s with the correct hand and then grasp the levers between finger and thumb in a precision grip. The lever position had to be maintained above a criterion displacement for 1 s before being released to obtain a food reward. Motors opposed lever movement, simulating the action of springs (force for initial lever movement: 0.15 N; spring constant: 0.03 N/mm). Movement of the incorrect hand or premature homepad switch release resulted in a failure tone and termination of that trial. In this report, we analyze only data from unimanual trials with the contralateral hand, relative to the side of the brain the data were recorded from, and for instructed delays of 1-s duration.

Animals had ad libitum access to water at all times. Food access was restricted during training and recordings but was ad libitum during the weekend. If the number of rewards taken during recordings fell below a threshold level for two consecutive days, animals were given ad libitum access to food on the second day. Between the start and end of the recording period (duration of 6 mo for *monkey E*, 11 mo for *monkey T*), both animals increased their weight (16% for *E* and 54% for *T*).

### Surgical Preparation

All surgical operations were performed under deep general anesthesia (2–2.5% isoflurane in 50:50 O_2_-N_2_O) and were followed by a full course of antibiotics [co-amoxyiclav 140/35, 1.75 mg/kg clavulanic acid, 7 mg/kg amoxicillin (Synulox); Pfizer] and analgesic [buprenorphine (Vetergesic), 10 μg/kg; Reckitt & Coleman, Hull, UK) treatment. In an initial surgery, epimysial patch electrodes were implanted over the following muscles bilaterally, with wires routed subcutaneously to a connector on the back: first dorsal interosseus (1DI), abductor pollicis brevis (AbPB), abductor pollicis longus (AbPL), flexor digitorum superficialis (FDS), extensor digitorum communis (EDC), biceps (Bic), and triceps (Tri). In a subsequent surgery, each monkey was implanted with a headpiece to allow atraumatic head fixation. Three recording chambers were also implanted to allow intracranial neuronal recordings to be carried out. A separate chamber was implanted over each M1 bilaterally (craniotomy center A18 and ML13), and a single chamber was implanted over the supplementary motor area (craniotomy center A20 and ML0), but those data will be presented in a subsequent report. Two insulated tungsten stimulating electrodes (LF501G; Microprobe, Potomac, MD) were chronically implanted in each pyramidal tract (PT) for antidromic identification of pyramidal tract neurons. The location of the electrode tips within the PT was verified histologically postmortem as described in our previous work (Soteropoulos et al. 2011, 2013).

### Neural Recordings

A 16-channel Eckhorn microdrive (Thomas Recording, Giessen, Germany) was used to make up to 14 simultaneous microelectrode penetrations into M1 during daily recording sessions (average number of electrodes used per session: 9, range: 3–14). Electrodes were platinum insulated with quartz glass and had a shaft diameter of 80 μm and impedance of 1–2 MΩ (Thomas Recording). Cells were identified as corticospinal if they responded at a constant latency to stimulation through the chronically implanted PT electrodes (up to 400 μA, 0.2-ms pulse, 1 Hz) and if the evoked spikes could be collided by orthodromic spikes occurring shortly before the stimulus. Single-unit activity (band pass, 300 Hz to 10 kHz, sampled at 25 kHz) was recorded while the animal performed the task, together with lever displacement, force, and EMG activity (band pass, 30 Hz to 2 kHz, sampled at 5 kHz). Off-line, action potential waveforms were discriminated to generate the occurrence times of single spikes with custom-written cluster-cutting software (Baker et al. 1998; Dyball and Bhumbra 2003). Only single units with a consistent spike waveform and no interspike intervals < 1 ms were used in subsequent analysis.

The hand representation of M1 was identified by multiple-pulse stimulation (13 biphasic stimuli, 0.2 ms per phase, 300 Hz train frequency, 1 Hz repetition rate) through the recording electrodes and visual observation of muscle twitches at low (<20 μA) current intensities.

### Data Analysis

#### EMG analysis.

For each recording session we normalized the rectified EMG activity to the peak value seen during the movement epochs of the task for each muscle separately. This allowed us to compare across sessions and animals whether EMG activity during the delay and movement periods was substantially modulated for either upper limb.

#### Spike-triggered averaging.

To identify cells with connections to motoneuronal pools from the recorded muscles, we carried out spike-triggered average (STA, ±2 s) analysis as described in our previous work (Soteropoulos et al. 2011). Briefly, for each cell snippets of rectified EMG activity aligned to the time of each spike were used to compile an average response for each muscle recorded. To overcome a nonstationary background in the STA (due to comodulation of muscle and cell firing), we estimated the baseline by convolving the STA with a Gaussian kernel of unit area and width parameter σ = 30 ms and then subtracted this from the STA. The standard deviation (SD) of this baseline-corrected STA was calculated, excluding the middle region within 50 ms of the triggering spike. To detect significant effects, the maximal and minimal values were found within a standard window 3–20 ms after spike. The number of bins within the 17-ms-long response region (total of 85 bins) that were larger or smaller than the 2-SD level was counted. The rest of the STA (excluding the middle ± 50 ms region) was subdivided into a total of 222 sections 17 ms long, and the same procedure was repeated. If the number of bins in the response region exceeding 2 SDs of the mean was larger than or equal to the maximum number found in the control region, this was considered a significant effect (*P* < 0.0045). All significant responses were further examined by recompiling the averages excluding sweeps with artifacts or other large changes in the EMG; only responses that were still visible in these averages are considered in results. Previously published criteria on the acceptable width of the effects were used (Baker and Lemon 1998) for a final selection of “causal” vs. “correlative” STA effects.

#### Neural activity analysis.

The times of spikes for single cells were aligned to the time of the GO cue signal for each trial (±4 s) with 1-ms bin width. The baseline firing rate for each cell was estimated as the mean number of spikes during the last 0.4 s of the homepad press at the start of the trial. For comparing responses across cells, as different neurons usually have different baseline firing rates, the responses were first converted to a *z* score. If we assume that the total number of spikes during an epoch of interest is a Poisson process, we can then determine whether the spike count is significantly different from a baseline epoch by calculating

(1) $$z=\left(\frac{N_{r}}{T_{r}}-\frac{N_{b}}{T_{b}}\right)/\sqrt{\left(\frac{N_{r}}{T_{r}^{2}}+\frac{N_{b}}{T_{b}^{2}}\right)}$$

where *N* corresponds to the total spike counts across *T* bins and the subscripts r and b correspond to the response and baseline epochs, respectively. The statistic *z* (Cope et al. 1987) can be treated as having a normal distribution with zero mean and unit variance, which can then allow testing of the probability that the response arose from a Poisson process with the same mean as that of the baseline epoch (see Equation 7 in Cope et al. 1987). *z* Values outside ±1.96 indicate that the response and baseline regions for a given cell are significantly different (*P* < 0.05). To estimate whether the response of a group of cells is significantly different from the baseline at a given time, the population *z* score (*Z*^) can be estimated by

(2) Z^=1N×∑i=1nzi

where *N* is the total number of cells and *z_i_* is the *z* score for the *i*th cell calculated with *Eq. 1* above. If the cell responses are drawn from a population with zero mean and unit variance, then summing *z* over all available cells and normalizing as in *Eq. 2*, *Z*^ should have zero mean and unit variance. This transform can be carried out at multiple time points relative to the baseline epoch, allowing us to produce a standardized perievent time histogram (zPETH). In addition, *Eq. 2* can be used to combine bins across an epoch as well as across cells, and in that instance *N* will correspond to the product of the numbers of cells and bins within that epoch.

To compare different cell groups unpaired *t*-tests were used accordingly on the *z* values (*Eq. 1*), or when there were multifactorial variables an ANOVA was used. The type of test used is stated next to the reported *P* values in results.

For part of the analysis it was desired to assess cell firing during movement with cell firing just before movement. To do this, the movement activity index (MI) was estimated, as described previously (Kaufman et al. 2013), as

(3) $$MI=\left(RT_{mov}-RT_{del}\right)/max\left(RT_{mov},RT_{del}\right)$$

where RT_mov_ corresponds to the mean firing rate relative to movement onset (±150 ms) and RT_del_ corresponds to the mean rate just before the GO signal (from −200 ms to 0 ms relative to the GO cue). Positive values mean that the rate was increased during movement relative to the preparatory epoch, and negative values show that there is rate suppression during movement. The index is constrained to have values from −1 to 1.

#### Regression analysis.

To assess the correlation of neural firing with reaction time, a simple linear regression analysis was carried out:

(4) $${\frac{1}{RT}}_{i}=\beta_{0}+\beta_{1}\text{×}\lambda_{i}+\varepsilon_{i}$$

where RT*_i_* and λ*_ι_* are the reaction time and firing rate of the cell for the *i*th trial and β_0_ and β_1_ are the constant and rate coefficients, respectively; ε is the residual error term. We estimated λ for each cell as the mean rate just before the onset of reach (−100 ms to +10 ms relative to the minimum reaction time for the trials available for that cell). To allow comparison of the rate coefficient β_1_ across various cells, the firing rate λ for each cell was normalized across trials by converting to a *z* score. We use 1/RT as a measure of reaction time, as this is a normalizing transform (a requirement of regression analysis) for skewed reaction time distributions. In addition, it allows for an intuitive presentation and discussion of the data—for an “excitatory neuron,” we would expect that when the cell fires more or sooner relative to the GO cue the reaction time will be smaller and hence 1/RT will be larger and produce a positive β_1_. For an “inhibitory cell,” we would expect that the reaction time will be greater when the cell is active and 1/RT will be smaller, which will instead return a negative β_1_.

It is worth noting that linear regression is used to give a measure of the directionality of the relationship of cell activity with behavior (as either positive or negative) and not as a model of neural firing. This is almost certainly likely to depend on many other linear and nonlinear interactions not included here.

## RESULTS

### Task Behavior

**Fig. 2.**
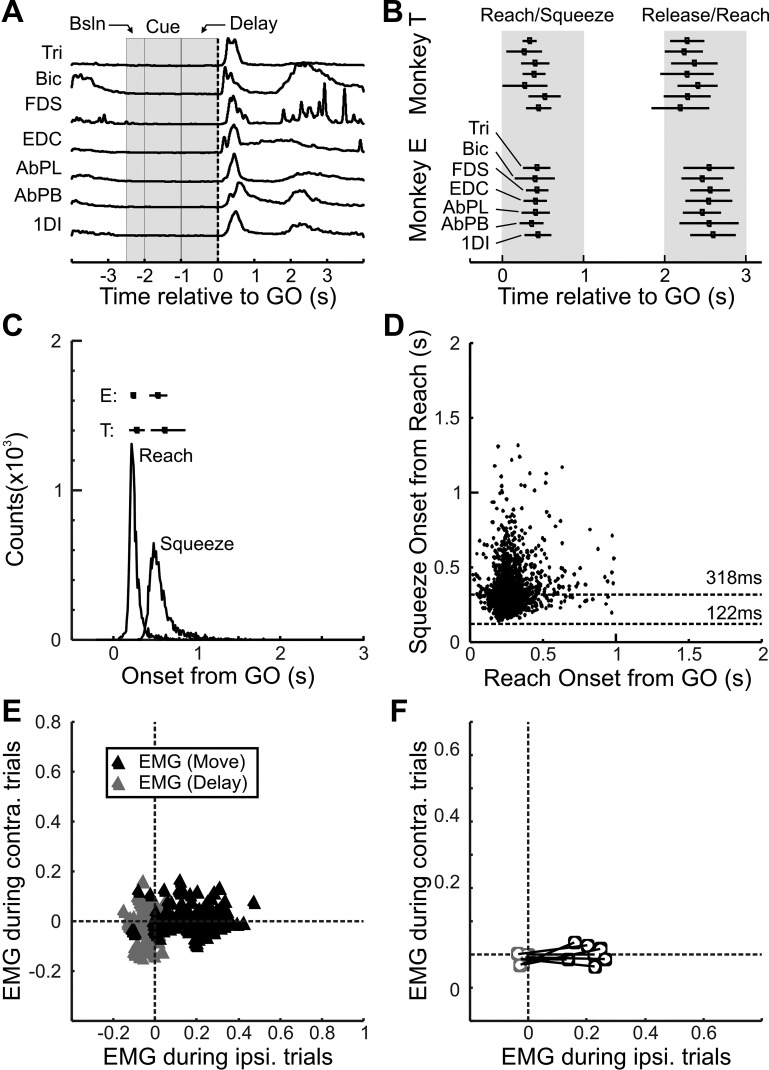
Task metrics and behavior. *A*: average EMG signals from recorded muscles in 1 session from *monkey E*, showing lack of EMG activity during the delay period until the GO cue. *B*: temporal distribution of peaks in rectified EMG responses of muscles in *monkeys E* and *T* during the reach to grasp and release and reach for reward parts of the trial. Boxes and horizontal lines indicate mean and 90% range of values for each muscle. *C*: distribution of reach onset and squeeze onset times. Boxes and horizontal lines at *top* indicate mean and range 90% of values for each animal for each event. *D*: cluster plot of reach onset relative to squeeze onset. Horizontal dashed lines indicate the minimum delay of squeeze onset from reach (122 ms) and the mean delay from reach (318 ms). *E*: cluster plot of mean EMG levels during movement and during the instructed delay during trials with the same (*x*-axis) and contralateral (*y*-axis) arms. *F*: same as *E* but showing the mean only for each muscle.

Both animals were able to perform the task correctly on most attempted trials (mean success rate of attempted trials per session was 91%, range 80–95%). Both animals kept both hands on the homepads for the duration of the cue and instructed delay periods, and reaching did not commence until after the GO signal (Fig. 2). The recorded upper limb muscles all showed a very similar pattern of activity (see Fig. 2*A*), with a burst of EMG shortly after the GO signal and then a reduced but maintained level of EMG during the grip, followed by another burst of EMG corresponding to the release of the levers and reaching for the food reward. The time of peak EMG for each muscle for each recording session was measured during the reach/grip and during lever release, and a summary of these data is shown in Fig. 2*B*. This pattern was similar across the two animals (Fig. 2*B*). For the onset of reach, the time at which the homepad the arm was resting on became unloaded was used as an estimate of the reaction time. For the onset of squeezing, the time at which either of the two finger levers was squeezed to 5% of its target distance was used. The distributions of the time for reach and squeeze relative to the GO cue are shown in Fig. 2*C*. The animals had comparable mean reaction times (*monkey E*: 237 ms, *monkey T*: 279 ms) and time of squeeze onset (*E*: 530 ms, T: 615 ms) relative to GO cue. Figure 2*D* shows a cluster plot of squeeze onset relative to reach onset, showing that on average squeeze onset occurred 318 ms after reach and no sooner than 122 ms. For a small fraction of trials (0.7%) the onset of the reaction time was <100 ms, which might represent the animal initiating the correct movement predictively rather than reactively.

For four recording sessions no EMG data were available because of a technical failure. However because intracortical microstimulation (ICMS) thresholds and PTN antidromic latencies were comparable to other penetrations, cells from these sessions were included in the database. As muscle activity from both arms was available, it was possible to test whether there was consistent modulation of EMG activity during the delay period for either arm. For each recording session the EMG activity was normalized relative to the peak of the mean EMG seen during the movement epoch for that muscle. Figure 2*E* plots the mean normalized EMG during the movement period (1 s after GO cue) for each muscle for each session, with the *x*-axis corresponding to the EMG during the trials ipsilateral to the reference muscle and the *y*-axis corresponding to the EMG during the contralateral trials. We also plot the EMG levels during the cue and instructed delay periods (2 s before GO cue). Most EMG levels during the delay period were clustered around the zero value, indicating that there was minimal EMG activity during the delay period. During the move period, the majority of EMG signals were clustered parallel to the *x*-axis, indicating that muscle activity was mostly lateralized during the movement. Figure 2*F* shows the mean for each muscle, which reconfirms what the cluster plot in Fig. 2*E* is showing.

### Neuronal Data

We recorded the activity of 211 neurons from the hand and arm area of M1. The ICMS thresholds from the electrodes where the cells were recorded were all <20 μA (bar one), with a mean threshold of 10 μA. For the purposes of the analysis used here cells were required to have >25 trials per trial laterality. This left us with 182 cells (89 cells from *monkey E* and 93 cells from *monkey T*) over multiple recording sessions (22 for *E* and 21 for *T*). The average number of trials for each cell was 139 (range 25–295). Just over half of the recorded cells (*n* = 110) were identified as PTNs (65 from *monkey E*, 45 from *monkey T*) as described in methods. These included mostly fast PTNs (94/110), with fast PTNs being cells with an antidromic latency ≤  2ms. The range of observed antidromic latencies was 0.8–4.7 ms, consistent with previous reports (Firmin et al. 2014; Vigneswaran et al. 2011). With spike-triggered averaging of muscle activity 16/110 PTNs were also identified as corticomotoneuronal cells (CM cells; 7 from *monkey E*, 9 from *monkey T*), and the STA metrics for these are presented in Table 1. All other cells were classified as unidentified (UID). It is important to clarify that the identification methods for PTNs and CM cells are not exclusive—identified cells are definitively PTNs or CM cells, but cells that fail to be identified as either type could still be PTN or CM cells.

**Table 1.** CM cell details

					Firing Rate, spikes/s			
Muscle	Onset, ms	PWHM, ms	MPI, %	No. of Spikes	Baseline	Cue	Delay	Move
AbPL	7	1.8	5.5089	27,364	2.8	3.9	8.0	15.1
AbPL	6	2.1	5.7433	79,866	9.8	12.3	18.0	25.5
FDS	6.8	1.3	0.5778	53,008	15.9	20.5	23.6	39.3
**BIC**	**5**	**4.0**	**1.175**	**79,995**	**33.3**	**33.3**	**31.6**	**28.5**
**EDC**	**6**	**5.0**	**3.3946**	**23,419**	**6.3**	**2.8**	**2.6**	**5.5**
**BIC**	**5.6**	**5.5**	**2.8029**	**79,989**	**21.9**	**21.1**	**17.5**	**25.5**
AbPL	7.8	3.9	1.9374	71,353	14.1	16.4	23.1	45.8
**EDC**	**6.2**	**6.5**	**2.8716**	**79,996**	**19.3**	**20.2**	**19.1**	**36.3**
BIC	7.6	2.5	1.4501	6,870	0.1	0.1	0.2	22.2
EDC	8	2.9	1.9519	14,868	1.8	1.7	3.4	19.5
BIC	6.8	4.1	1.6011	18,092	0.3	1.2	0.8	30.3
BIC	5	5.5	2.6162	17,336	2.8	3.1	4.1	0.8
FDS	7	5.5	1.2574	79,978	25.8	26.4	26.9	43.8
FDS	7	6.2	1.1825	79,985	24.5	23.4	24.7	32.6
EDC	7	5.5	1.884	54,534	6.9	10.6	17.3	54.2
EDC	5.2	5.8	1.8115	74,549	17.6	20.7	23.0	59.6

First column shows muscle with largest STA effect from the cell; 2nd column shows onset latency of STA effect; 3rd column shows peak width at half-maximum (PWHM); 4th column shows mean percentage increase (MPI) of EMG during STA effect; 5th column shows no. of spikes for each cell. Next 4 columns show mean firing rate for the different task epochs as defined in text. Cells that showed a rate suppression during the delay period compared with baseline are in bold. Only cells with STA effects considered causal are shown.

#### Analysis of firing rates.

**Fig. 3.**
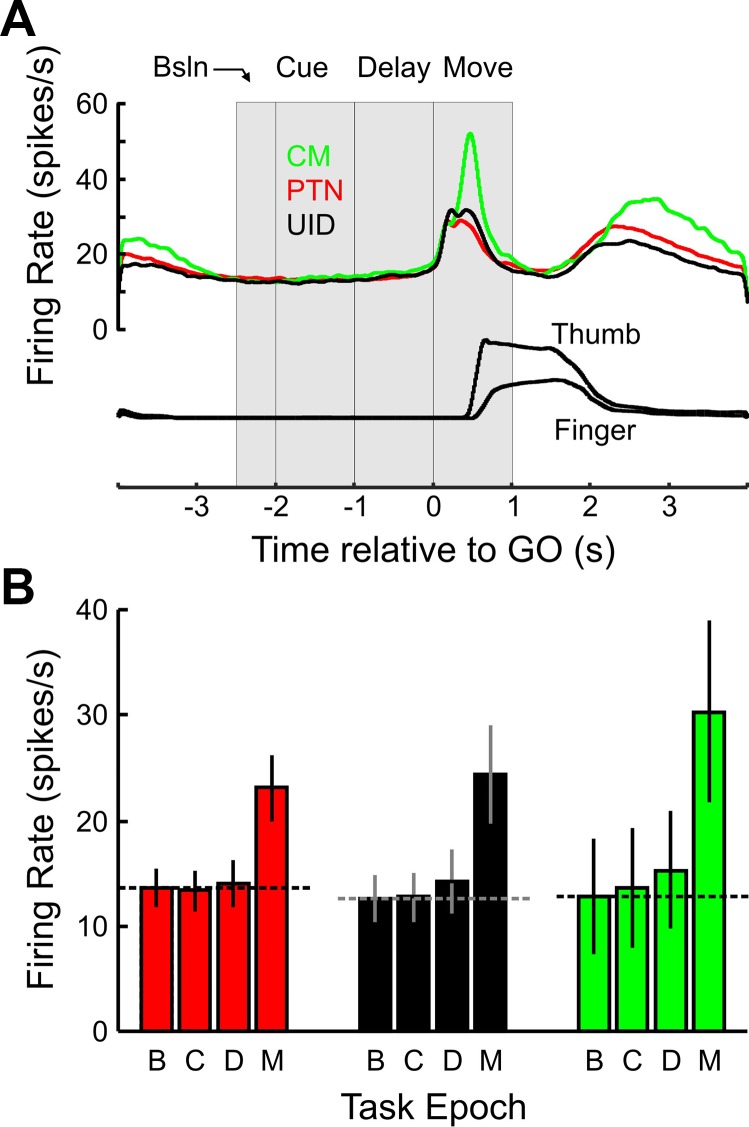
Firing rate responses of neurons. *A*: *top* traces show the mean firing rates of PTNs, CM cells, and UIDs as a function of time relative to the GO cue. Traces have been smoothed by convolving with a Gaussian kernel of unit area and σ of 30 ms. Vertical gray bars delineate the task epochs that were used to analyze mean firing rates. *Bottom* 2 traces show representative traces of lever position signals for the index finger and thumb. Color code for cells applies to *B*. *B*: population responses across different cell types during baseline (B), cue (C), instructed delay (D), and move (M) epochs. Bars correspond to the mean firing rate of the cell population for each epoch. Error bars correspond to the 95% confidence intervals for the mean rate for each epoch; dashed lines correspond to the mean baseline rate for the given cell group.

PETHs were generated (1-ms bin width) with neural activity aligned to the GO cue, and the mean rate of the population of recorded cells was measured. This is summarized in Fig. 3. The mean activity profiles for the different cell types are shown in Fig. 3*A*. Neural activity during the last 0.4 s of the homepad press period, when there was minimal EMG activity, was variable and ranged from 0 to 37 spikes/s (mean 13.1 Hz). This epoch comprised the “baseline” epoch for this and all further analyses. There was no significant difference in the baseline firing between the different cell populations (*P* > 0.4, *F* = 0.77, 1-way ANOVA). Comparison of activity during the cue presentation (−2 to −1 s before the GO cue), during the instructed delay period (−1 s before and up to the GO cue), and during the movement period (0–1 s after the GO cue) similarly revealed no significant differences between the cell groups (1-way ANOVAs, all *P* values > 0.05). Figure 3*B* shows the mean firing rate for the different epochs for the different cell types. There was a significant increase in firing during the movement epoch for all cell types but not so for the cue and delay periods. Based on the mean population firing rates alone, there was no evidence for any suppression in neural activity during the delay period or any difference between the different cell groups.

#### Analysis of normalized neural activity.

**Fig. 4.**
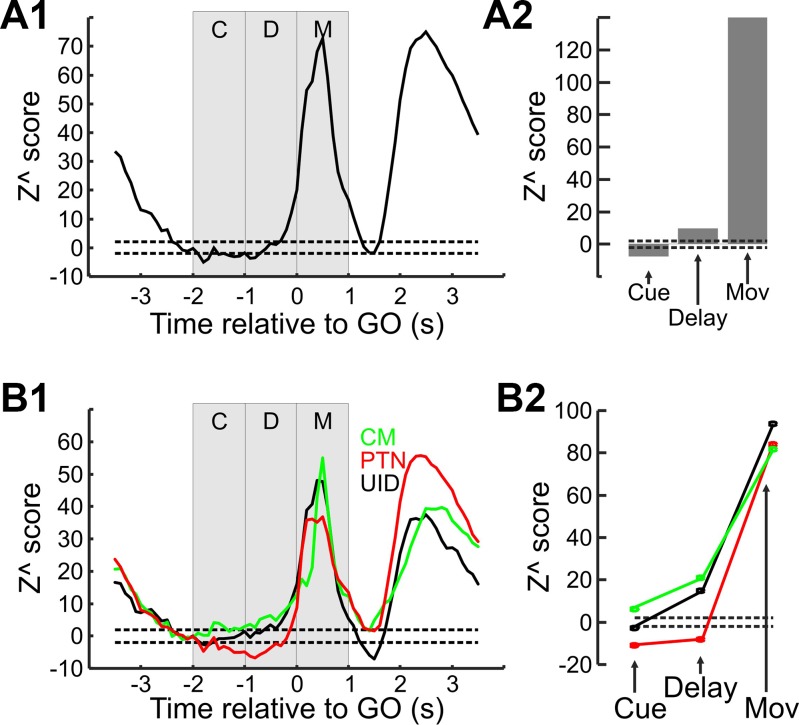
Standardized responses of neurons. *A*: normalized responses of all recorded cells. *A1*: population *Z*^ score of all cells across time during the task. The zPETH was constructed with 100-ms nonoverlapping windows. Vertical gray bars delineate the times used for further epoch analysis—cue (C), instructed delay (D), and move (M). Note the significant suppression (*Z*^ < −2) during most of the cue and delay epochs. *A2*: population *Z*^ score across all cells for the corresponding epochs showing significant suppression during the cue presentation and significant facilitation during the movement epoch but not during the delay period. *B1*: same as *A1*, but different colors correspond to different cell populations (PTN, CM, and UID); the same color code applies to *B2*. *B2*: population *Z*^ score across the difference cell types for the different epochs.

To be able to compare neural firing across cells with different background rates, cell activity was first converted to a *z* score as described in methods (*Eqs. 1* and *2*). Figure 4*A1* shows the population *z* score across all cells at various time points during the task in 100-ms nonoverlapping windows (zPETH). Values less than −2 indicate a significant suppression of neural activity (*P* < 0.05), and as can be seen from Fig. 4*A1* there was significant suppression during most of the cue and instructed delay periods. The same analysis across the 1-s epochs (Fig. 4*A2*) showed that the suppression is highly significant during the cue period (Z^ = −7.74, *P* < 0.00001). During the delay period at the population level there was significant facilitation (Z^ = 9.7). At the population level there is a very highly significant level (*P* < 0.000001) of facilitation relative to baseline during movement.

To determine whether the different cell types showed different levels of delay suppression the same analysis was carried out for the different cell types, and these results are shown in Fig. 4*B*. Figure 4*B1* shows the zPETH for PTNs, CM cells, and UIDs separately. We can immediately see some differences between the different neuronal subtypes during the cue and delay epochs. There is significant suppression for PTNs, but for UID and CM cells there is mostly facilitation instead. This is summarized by the epoch *Z*^ scores shown in Fig. 4*B2*, where PTNs were significantly suppressed during both cue (*Z*^ = −10.8, *P* < 0.0001) and delay (*Z*^ = −8.1, *P* < 0.0001) epochs; UIDs were suppressed only during the cue period (*Z*^ = −2.8, *P* < 0.0001) while significantly facilitated during the delay period (*Z*^ = 14.8, *P* < 0.0001). CM cells are significantly facilitated during both cue (*Z*^ = 6.2, *P* < 0.035) and delay (*Z*^ = 20.8, *P* < 0.0001) epochs. The facilitation during the movement epochs was highly significant across all cell types (*Z*^ > 80, *P* < 0.000001).

There were also significant differences between the cell types during the different epochs (1-way ANOVA test for bins in each epoch, in all cases *P* < 0.00001). During the cue and delay periods, all cell types were significantly different from each other, in the order of PTNs < UIDs < CM cells (*P* < 0.05, Tukey-Kramer adjusted for multiple comparisons). During the movement period, CM cells had significantly higher responses than either PTNs or UIDs (*P* < 0.05, Tukey-Kramer adjusted for multiple comparisons), but PTN and UID responses did not differ.

#### Reproducibility and validation of delay suppression.

**Fig. 5.**
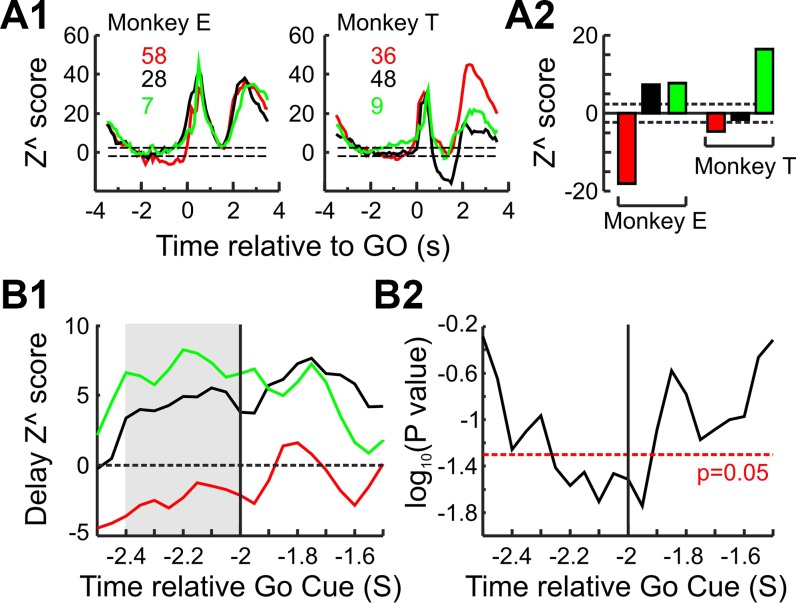
Reproducibility and robustness of *Z*^ score measures. *A1*: zPETH for the different cell populations constructed separately for each animal. Colored numbers within each subplot correspond to the number of cells for each type; color code applies to rest of figure (red for PTNs, green for CM cells and black for UIDs). *A2*: population *Z*^ score for the different cell types and animals during the cue and delay periods. For *A1* and *A2*, dashed lines correspond to ±2*z* value, which corresponds to a *P* value of 0.05. *B*: sensitivity of suppression during the delay to the time chosen for the baseline epoch. *B1*: value of delay *Z*^ score for the different cell types as baseline epoch (100 ms) is moved from −2.5 s up to −1.5 s relative to the GO cue. The onset of the laterality cue occurs at −2 s. *B2*: *P* value for ANOVA test of delay *Z*^ score and cell type for traces shown in *B1*.

To ensure that the delay suppression seen in the corticospinal system was present in both animals the same analysis was repeated separately for each animal, and the results are shown in Fig. 5. Figure 5*A1* shows the zPETH for each cell type in each animal. Although suppression of the corticospinal system is more pronounced in *monkey E* during the cue and delay periods, it is also observed for *monkey T*. Figure 5*A2* shows the mean population Z^ score across the cue and delay periods combined for each animal. The results of the two animals are comparable: PTNs are significantly suppressed during the delay period, while CM cells showed a significant facilitation during the combined delay epoch. An unpaired *t*-test found no significant difference (*P* > 0.2) between the two animals when comparing the epoch *Z*^ values during the cue and delay periods combined. The UIDs *Z*^ scores are different between the two animals, but as there is no way of knowing whether the UIDs in one animal are comprised of a different mix of various cell types compared with the other, the difference is not necessarily surprising.

The level of rate suppression during the delay period depends on the rate estimate during the baseline period. Most trials were carried out with little delay between them, so just before the homepad press some trials had substantial EMG and cell activity relating to the previous trial. As it is possible that not all EMG and cell activity had fully returned to resting levels, if the baseline rate was overestimated then it is possible that there is also an overestimate in the rate suppression. Furthermore, if the firing rate of the different neural populations does not return to baseline at the same time then the difference between the cell groups shown in Fig. 4 could also be artifactual. To test for this, the level of suppression during the delay period was recalculated for each cell group with a sliding baseline window of width 100 ms (in 50-ms steps), from the start of the homepad press (−2.5 s from GO cue) up to 0.5 s into the laterality cue, and this is shown in Fig. 5*B*. Figure 5*B1* shows the *Z*^ score for the different cell types. *Z*^ values in all cell types are more negative at the very start of the homepad press, probably reflecting the rate of some cells not returning back to baseline. However, even when using baseline epochs much closer to the onset of the laterality cue, the rate suppression seen in PTNs during the delay period remains, as does the relative relationship between the different cell types—regardless of which bin is chosen to estimate the baseline rate, PTN firing is always lower than that of CM cells and UIDs during the delay period. Figure 5*B2* shows the significance level of a one-way ANOVA carried out for the data shown in Fig. 5*B1*. There is a significant difference in the delay firing between the three types of cells for several time points just before the onset of the laterality cue.

**Fig. 6.**
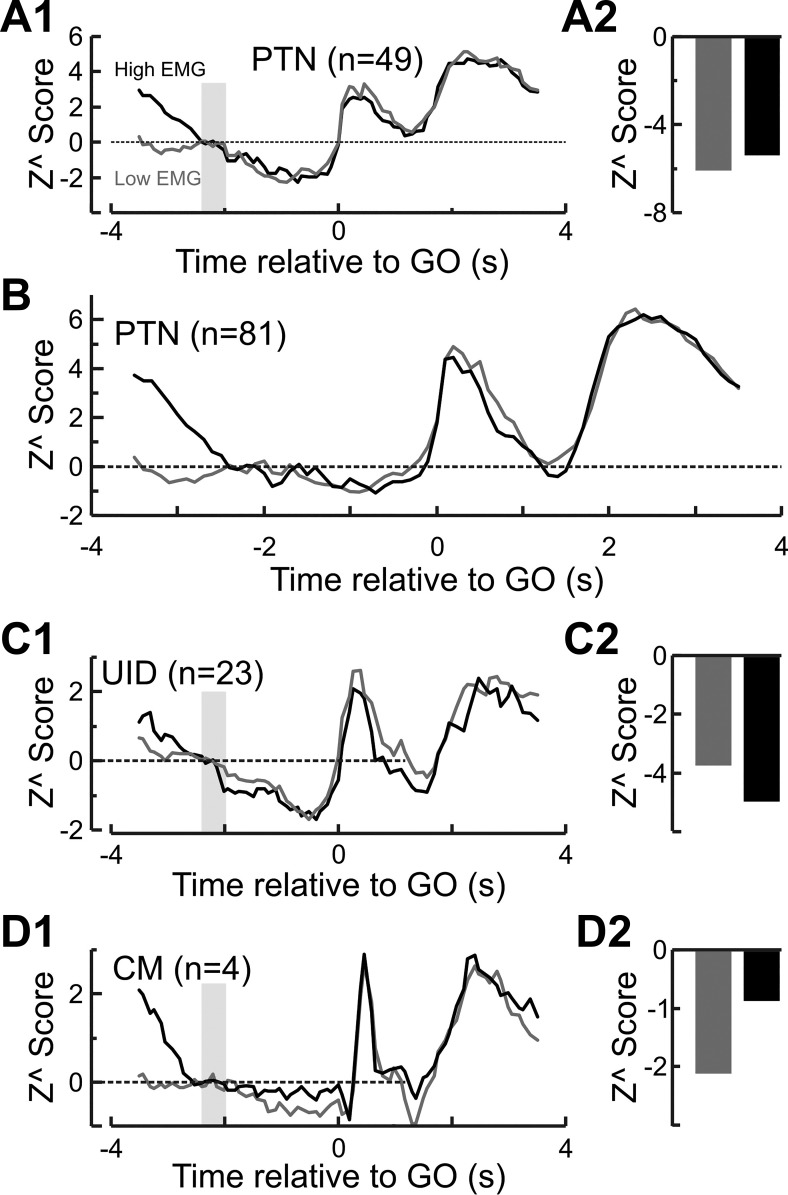
Influence of pretrial EMG activity on delay suppression. *A1*: zPETH for PTNs using trials with high (highest 33%) and low (lowest 33%) levels of EMG before the onset of the homepad press. Only cells that showed rate suppression during the delay period using all trials were included. *A2*: epoch *Z*^ score during the cue and delay periods for the zPETHs shown in *A1*. There was no significant difference (*P* > 0.5, paired *t*-test) in the delay suppression. *B*: same as *A1* but using all available PTNs. Number of PTNs is smaller than the total number available as cells recorded during sessions with no EMG activity were excluded from this analysis. *C*: same as *A* but for UIDs. There was no significant difference (*P* > 0.16, paired *t*-test) between the *Z*^ scores for trials with high and low background EMG. *D*: same as *A* but for CM cells. Suppression of firing during the cue and delay periods was significantly greater (*P* < 0.001, paired *t*-test) for trials with the lowest level of background EMG.

A further test was carried out to confirm that the delay suppression was not an artifact of the chosen baseline period. For each trial the mean level of EMG just before the baseline period (−3.5 to −2.5 s relative to GO cue) was measured for all muscles and normalized to a *z* score within each muscle. This was averaged across muscles to generate an estimate of the mean EMG activity across muscles for a given trial. The top 33% and lowest 33% of trials were used to regenerate a “high-EMG” and “low-EMG” zPETH for cells. If the rate suppression was an artifact due to residual cell activity from the previous trial then we would expect it to be abolished or reduced for “low-EMG” trials. The results of this analysis are shown in Fig. 6.

Figure 6*A* shows the zPETH for high- and low-EMG trials for PTNs that showed suppression during the delay period. Relative to baseline there is no substantial difference in the level of suppression during the delay period, and this is borne out by the mean epoch *Z*^ scores (high EMG: −5.44, low EMG: −6.2; *P* > 0.5, paired *t*-test) shown in Fig. 6*A2*. Figure 6*B* shows the zPETH for low- and high-EMG trials using all PTNs, and again there is no major difference between the two. Figure 6*C* is the same as Fig. 6*A* but for UID cells—as with PTNs there was no significant difference between high- and low-EMG trials (high EMG: −4.3, low EMG: −3.9; *P* > 0.16, paired *t*-test). Figure 6*D* shows the same for the CM cells, and in this case there is a significant difference (*P* < 0.001, paired *t*-test) but in the opposite direction than expected. The level of suppression is larger for low-EMG trials than for high-EMG trials (high EMG: −1.1, low EMG: −2.3).

**Fig. 7.**
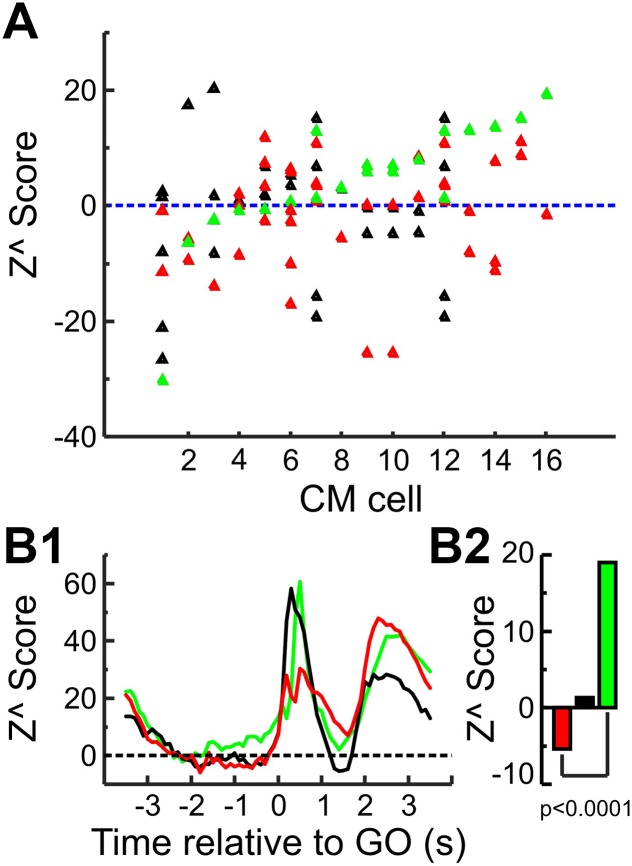
Comparison of PTN and CM delay suppression. *A*: epoch *Z*^ score during the delay period for CM cells, PTNs, and UIDs recorded within the same session (green, CM; red, PTN; black, UID). Color code applies to entire figure. Each triangle corresponds to a single neuron, and the data are plotted and ordered by the *Z*^ value of the given CM cell within the recording session. Note that as 2 sessions had 2 CM cells recorded at the same time, these sessions appear twice in the plot. *B*: mean results but using only cells recorded within the same session as the CM cells. *B1*: population *Z*^ score of the different cell types. Note that there is still suppression of firing in PTNs compared with CM cells. *B2*: epoch *Z*^ score for cue and delay periods combined for different cell types—note significant difference between CM and PTN cells (*P* < 0.0001, unpaired *t*-test).

Not all recording sessions included CM cells as part of the recorded cohort. It is thus possible that the difference between CM cells and PTNs is related to some difference between these recording sessions, either in the monkey performance of the task or the location of the recordings in M1. To check whether that is the case, a comparison was made between the different cell types using only cells recorded from sessions with CM cells—this reduced data set consisted of 35 PTNs, 25 UIDs, and the original 16 CM cells from 14 recording sessions. The results are shown in Fig. 7. Figure 7*A* shows the individual *Z*^ score values during the cue and delay periods combined for each cell type. The CM cells are sorted on the basis of their *Z*^ score values. As can be seen from this figure, many PTNs had lower *Z*^ score values than CM cells. This is borne out by the population averages shown in Fig. 7*B*. Figure 7*B1* shows the mean zPETH for the different cell types, while Fig. 7*B2* shows the mean *Z*^ score for the cue and delay periods combined. As PTNs recorded in the same sessions still show rate suppression (cue and delay epoch *Z*^ score: −5.6) compared with the rate facilitation shown by CM cells (cue and delay epoch *Z*^ score: 18.9), the difference between the two cell types cannot be attributed to any intersession differences.

To summarize, as a population corticospinal cells across both animals showed a significant suppression in their activity during both the cue presentation and the delay period, and this was less consistent for UIDs, while for CM cells this effect was instead a significant facilitation of neural firing.

### Relationship Between Delay Rate Suppression and Movement Onset

If the rate suppression during the delay period has a functional role to play in withholding action until the GO signal, it should be weaker for trials with a faster reaction time compared with those with a slower reaction time.

**Fig. 8.**
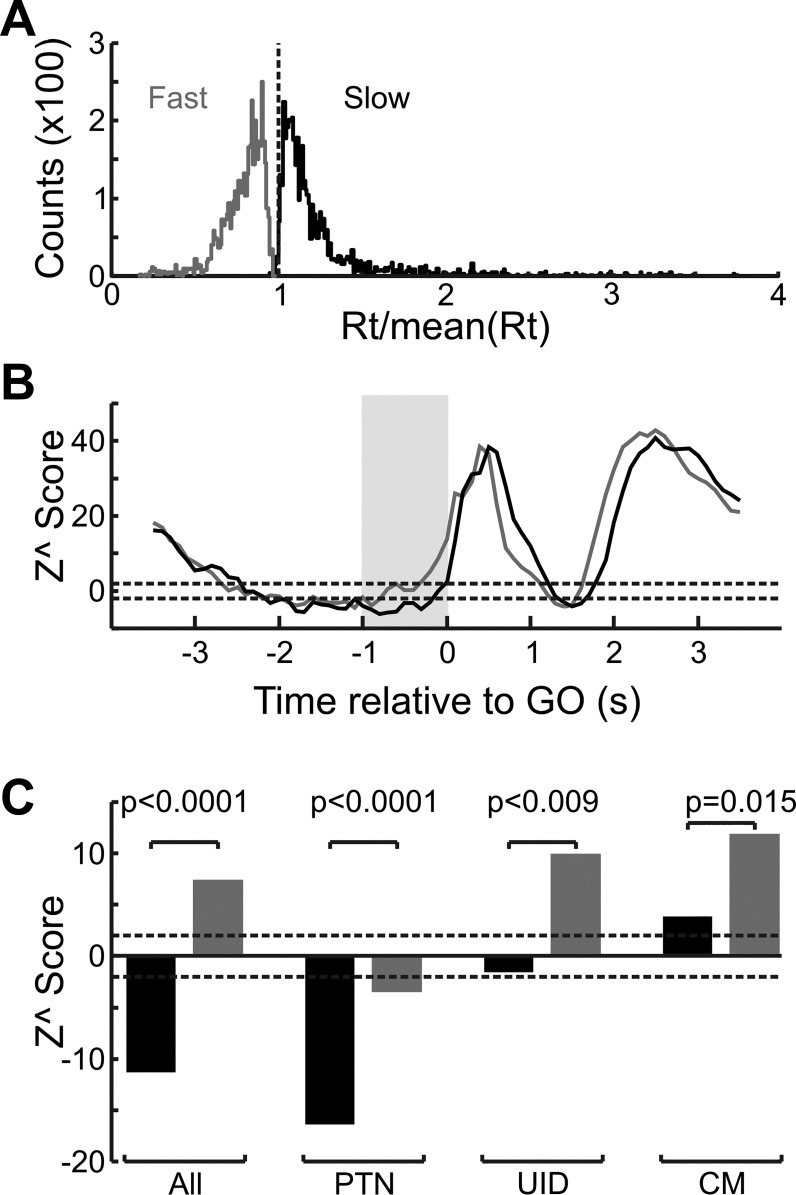
Delay suppression and reaction time. *A*: distribution of reaction times (Rt) for fast and slow trials. Reaction time is expressed as a fraction of the mean reaction time for the given recording session per cell. Color code applies to entire figure. *B*: population zPETH for slow vs. fast trials for all cells. Gray box indicates epoch that was used to generate *C*. *C*: population *Z*^ scores for the different cell types during the instructed delay period. For all subplots, dashed lines correspond to ±2*z* value, which corresponds to a *P* value of 0.05. *P* values over each pair of bars are *P* value of a paired *t*-test comparing the *Z*^ score for fast vs. slow trials.

To test for this, for each neuron the slowest 33% and the fastest 33% of trials were used to recompile the normalized neural responses (as described in methods, *Eq. 1*). The results are shown in Fig. 8. Figure 8*A* shows the histogram of normalized reaction times for the fastest 33% and slowest 33% of trials. Instead of plotting the raw reaction times in milliseconds (as in Fig. 2*C*), they were expressed as a fraction of the mean reaction time of the trials available for the given cell. As can be seen from Fig. 8*A* there was minimal overlap between the two distributions. Figure 8*B* overlays the zPETHs for fast vs. slow trials across all cells. During the cue delay there was a significant suppression for both fast and slow trials (*Z*^ = −9.3 and −7.2, respectively, *P* < 0.00001 for both). For the instructed delay period, the suppression remained for the slow trial zPETH (*Z*^ = −7.8, *P* < 0.00001) but not so for the fast trials—most bins are > −2 value and some are even larger than the *Z*^ = 2, showing significant facilitation. This was borne out by the population data, as the population *Z*^ score during fast trials (Fig. 8*C*) was 10.1 and significantly positive (*P* < 0.00001). The different cell types recorded from showed the same pattern, whereby there was less suppression for fast trials compared with slow trials. For PTNs and UIDS there was a highly significant difference between fast and slow trial conditions (paired *t*-test, *P* < 0.001 for both). For PTNs there was a very strong suppression for slow trials (*Z*^ = −14.7, *P* < 0.00001) whereas for UIDs this was not significantly different from baseline (*Z*^ = 0.66, *P* > 0.05), but for fast trials UIDs showed a very significant facilitation of their activity relative to baseline (*Z*^ = 11.9, *P* < 0.0001) whereas PTNs still showed a suppression, albeit a significantly weaker one (*Z*^ = −2.8, *P* < 0.00001). Although CM cells showed facilitation during both slow and fast trials, there was significantly less (*P* < 0.015) facilitation during slow trials (*Z*^ = 7.98, *P* < 0.00001) compared with fast trials (*Z*^ = 15.43, *P* < 0.00001). The significance value for CM cells is just less than the Bonferroni-adjusted significance level (*P* = 0.01666) for multiple comparisons.

To summarize, population neural activity was more suppressed (or less facilitated) during slow trials compared with fast trials, which is the expected result if the suppression was related to withholding a movement. This effect was consistent across cell types.

### Rate Suppression During Movement

The expectation is that even if corticospinal cells show suppression in firing during the delay period they should show an increase in firing rate during movement, and the inverse pattern would be seen for inhibitory cells (Kaufman et al. 2013). This prediction can be directly tested with this data. As UIDs are likely to contain an unknown mix of pyramidal and inhibitory interneurons it is not possible to make a clear prediction for that group.

**Fig. 9.**
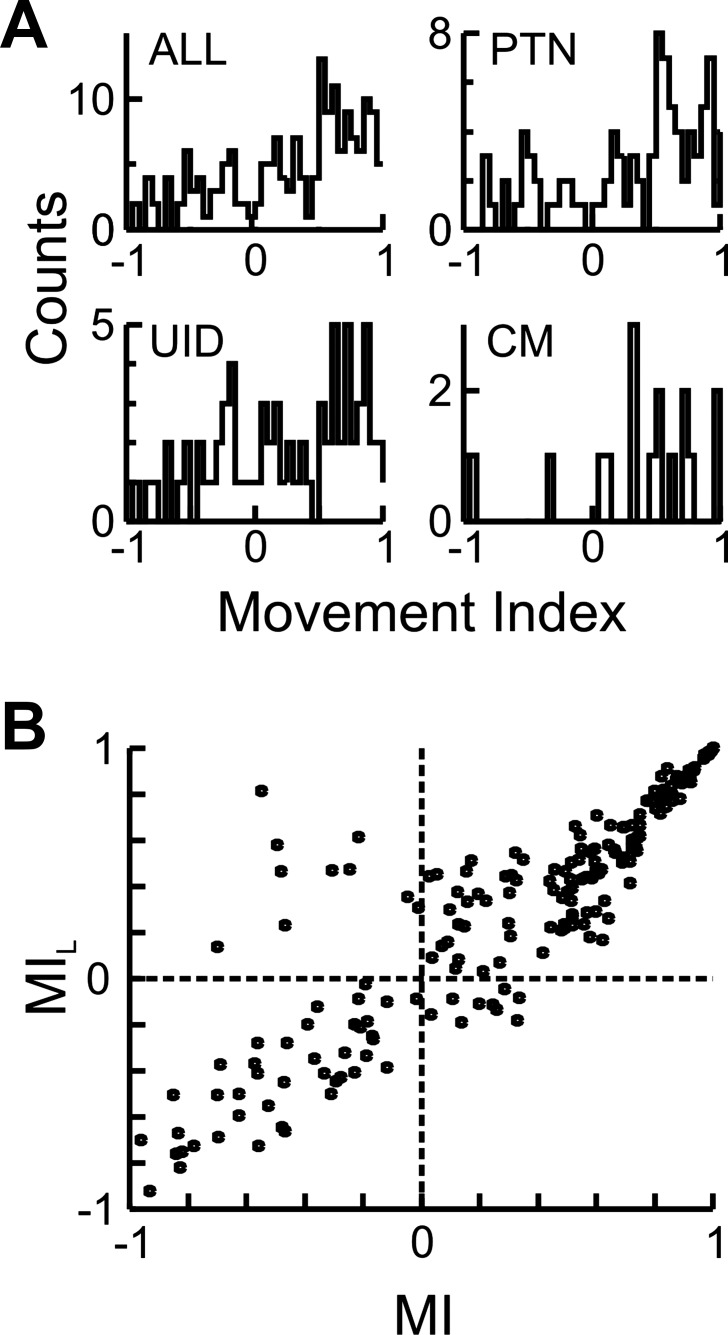
Movement index distribution. *A*: MI distribution for all cells and the different cell types. *B*: cluster plot of MI estimated as defined in *Eq. 3* and of a MI estimated by using the 1-s period after the GO cue to estimate the rate (MI_L_). Note that most cells with a negative MI also show a negative MI_L_, showing that the rate suppression during movement is not only limited to the perimovement epoch.

**Fig. 10.**
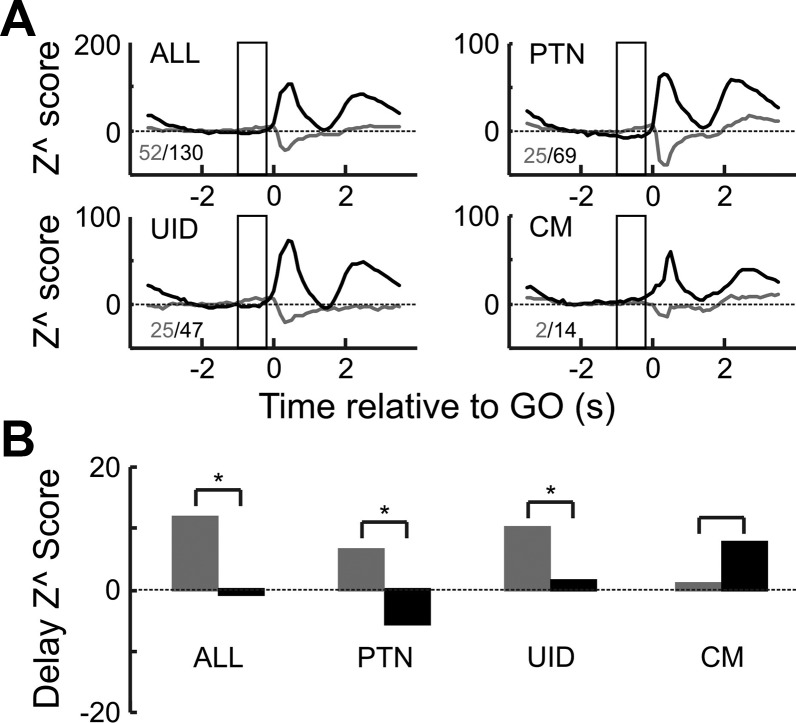
Population responses of cells with different movement indexes. *A*: zPETH of different cell populations. zPETH responses of cells with a positive MI are in black, and zPETH responses of cells with a negative MI are in gray. Numbers within each subplot indicate the number of cells in each MI category. Box highlights the epoch (−1 to −0.2 s relative to GO cue) used to estimate the values in *B*. *B*: epoch *Z*^ score for different cell types. *P < 0.003, unpaired *t*-test.

Some of the analysis already done (Figs. 3 and 4) has confirmed that at the population level both PTNs and CM cells show increased firing during movement, but it is not clear how ubiquitous that is for all the recorded cells. Therefore, the rate during movement onset was compared to that just before the GO signal (see *Eq. 3*, methods) and the results are shown in Figs. 9 and 10.

Figure 9*A* shows the distribution of the MI values for the three different cell types. The mean index value was positive (PTN: 0.3, UID: 0.23, CM: 0.37) for all cell groups and significantly (*P* < 0.05, *t*-test) larger than zero. Even though there are more cells with positive values than negative, there was a substantial fraction of cells for which the rate was suppressed around the time of movement onset (PTN: 27%, UID: 35%, CM: 12%). As the epoch used in determining the MI was focused on the time of movement onset, it is possible that cells with a negative MI only showed a transient suppression in firing rate around that time, and could later have had an increase in rate. To test for this, the MI was reestimated but using the mean rate during the entire 1 s after the GO cue, and this is plotted vs. the previous index in Fig. 9*B*. This reveals that most cells with a suppression during movement onset also showed suppression for most of the movement epoch, as very similar fractions of cells showing a rate suppression (PTN: 28%, UID: 29%, CM: 18%).

Figure 10 shows the mean zPETHs for cells with positive and negative MIs. A notable observation is the response of cells during the delay period. PTNs and UIDs that showed a rate suppression during movement (i.e., that behaved as “inhibitory” cells) showed no suppression (or instead facilitation) during the delay period. In contrast, cells with an “excitatory” profile during movement showed a lower rate during the delay period. This does not have to be the case—for example, for the two CM cells with an “inhibitory” profile (Fig. 10*A*), the rate during the delay period is lower than that for the cells with an excitatory profile. Although as a population identified excitatory cells within M1 show a response profile predicted by the gating model, a substantial minority show a response profile that would be expected from “inhibitory” cells, and this is particularly so for PTNs. Figure 10*B* shows the delay epoch *Z*^ scores for the cells with positive and negative MIs. For PTNs and UIDs the Z^ scores were significantly (*P* < 0.003) more positive for cells with negative MIs than those with positive MIs (PTN: 6.6 vs. −5.5, UID: 10.2 vs. 1.5). For CM cells the inverse pattern was seen (1.1 vs. 7.8) but was not significant, probably as only 2/16 cells showed a negative MI.

This analysis shows that although as a population corticospinal cells in M1 behave as predicted from M1 gating models, a substantial minority deviate from the prediction and show suppression of firing during movement and maintained activity during the delay period.

### Rate Correlation During Movement

Instead of comparing the mean rate during movement relative to the delay period to characterize a cell firing with movement, another approach would be to look at how cell firing is correlated with behavior on a trial-by-trial basis. Neural activity that is responsible for driving or withholding movement should at least show some degree of correlation with variability in reaction time, and the direction of this relationship can be used to infer whether a cell should be classified as “negative” or “positive” with respect to behavior. This was tested by using a linear regression model of neural firing with the reaction times on a trial-by-trial basis as described in methods (*Eq. 4*).

**Fig. 11.**
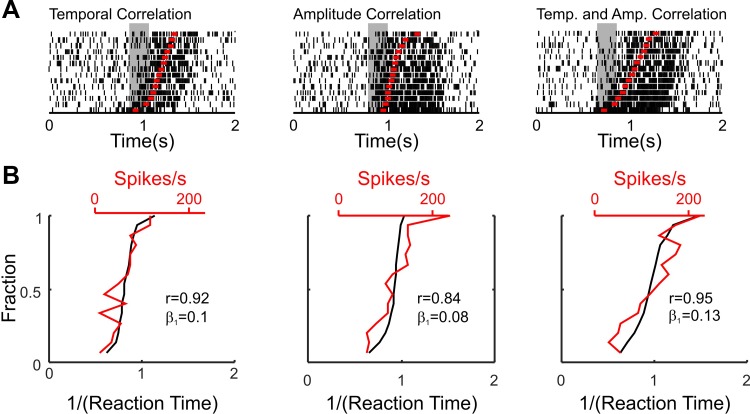
Simulated data for temporal and amplitude correlation of neural firing with reaction time. *A*: raster plots showing spiking for simulated neurons with a rate increase in firing rate. The time of the simulated movement onset for that trial is in red. Gray box indicates period used for rate estimation. *Left*: cell with a temporal correlation of firing with movement onset. *Center*: cell with amplitude correlation of firing rate during the step with movement onset. *Right*: cell with both amplitude and temporal correlation. *B*: sorted reaction times (1/reaction time, black) and the corresponding estimate of the firing rate for the given trial (red). Each plot corresponds to the raster shown in *A*.

This relationship could occur in the temporal domain, such that movement onset is correlated to when the neuron responds (but the amount the cell fires before movement is the same from trial to trial). The relationship could also occur in terms of the amplitude of the response—movement onset would be correlated with how much the neuron fires. The two are not mutual exclusive possibilities, and Fig. 11 shows this with simulated data for a cell with a positive relationship with movement onset. Figure 11*A* shows raster plots of three simulated neurons, with firing rates based on rate step processes with underlying Poisson statistics (Soteropoulos and Baker 2009); for the left column the response consisted of a rate step from 20 Hz to 100 Hz, but with the onset time of the step jittered (normal distribution with σ = 100 ms). The red marks indicate the “movement” onset. For the simulated cell in Fig. 11, *center*, the onset of the same rate step was constant from trial to trial, but in this case the amplitude of the step was variable (mean of 100 Hz, σ = 100 Hz). Finally, the simulated cell in Fig. 11, *right*, shows both types of correlation combined. The gray window indicates the epoch over which the firing rate was used for the regression analysis. Figure 11*B* shows how the sorted reaction times correlated with the estimated firing rate over the chosen epoch; the plot in black shows the sorted reaction times while the corresponding firing rates are in red (axis for firing rate is shown at top). Whether the correlation of neural firing occurs in the temporal or amplitude domains (or both), the regression coefficient for neural firing is positive.

For “positive” cells we would expect this correlation to be positive—the sooner or more the cell fires relative to the GO signal, the sooner the movement would be initiated—but no assumption is made about a baseline period. For “negative” cells the expectation is that this correlation will be negative—the longer or the more the cell fires relative to the GO signal, the slower the movement onset—but does not explicitly require the cell to show rate suppression during movement relative to any baseline.

**Fig. 12.**
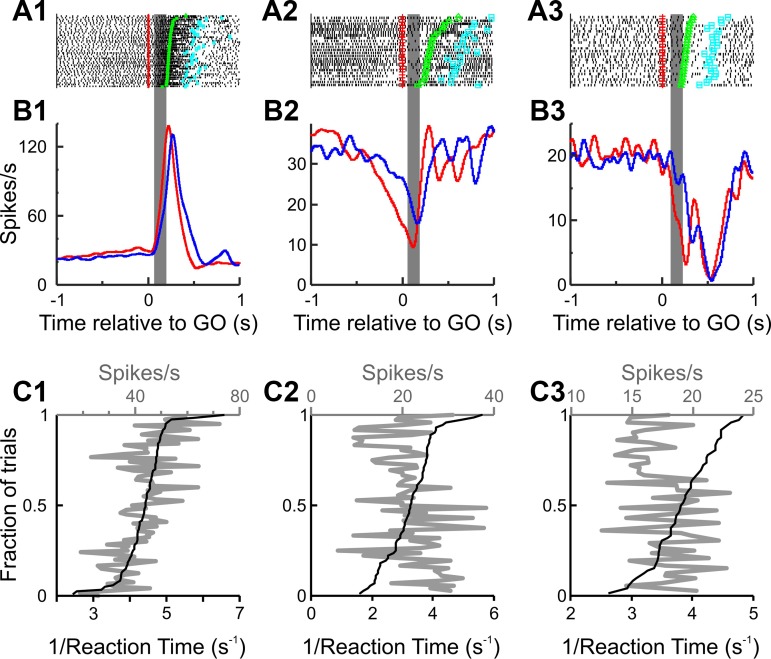
Exemplar cells and regression analysis. Each column corresponds to one PTN. *A*: raster plot in which each row corresponds to a single trial and the dark dots correspond to times of single action potentials. Red crosses correspond to the time of the GO cue, green triangles correspond to the time of movement onset for each trial, and cyan squares indicate the time of squeeze onset. The trials have been sorted by reaction time. Gray box indicates the period used for rate estimation. *B*: mean response of each cell for fastest 33% (red) and slowest 33% (blue) of trials. *C*: plot of sorted reaction times (as 1/reaction time, black) and the rate for the corresponding trial (gray).

Figure 12 shows this approach applied to three PTNs that showed a significant (*P* < 0.05) correlation with reaction time. Figure 12*A* shows the raster plots for each cell, where black lines correspond to the spikes fired by the cells, the GO cue is in red, the movement onset is in green, and the onset of the lever squeeze is in cyan. Figure 12*B* shows the mean PETH for the cells for fast (fastest 33%) and slow (slowest 33%) trials with regard to movement onset. Figure 12*C* shows how the rate of the cells was correlated to the time of movement onset (in the same fashion as shown for Fig. 11). For the first cell the correlation between 1/reaction time and firing rate is positive—the more the cells fire at a given trial relative to the GO signal, the smaller the reaction time. The other two PTNs show suppression in firing around movement onset, but in both cases the correlation structure is negative and these would be classified as “negative”—the less the cells fire on a given trial, the sooner the movement occurs.

**Fig. 13.**
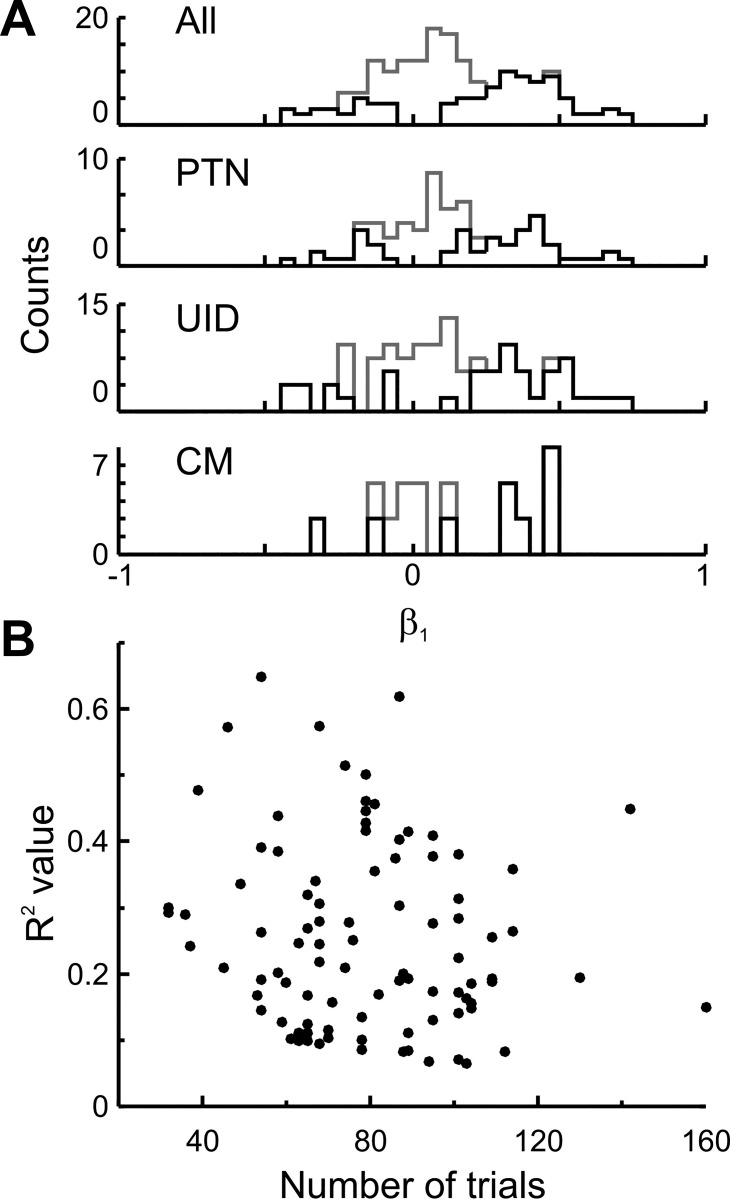
Regression and correlation coefficients. *A*: histogram of regression coefficient (β_1_) for all cells and different cell types. Gray, histograms of all cells; black, histograms for cells with significant (*P* < 0.05) correlation. *B*: cluster plot of *R*^2^ values relative to the number of trials per cell with significant correlation. Note that as the number of trials is reduced, the minimum detectable *R*^2^ increases.

The distribution of the regression (*R*^2^) and correlation (β_1_) coefficients for cells with significant correlation (*P* < 0.05) is shown in Fig. 13. If PTNs and CM cells are responsible purely for driving movement (and motoneurons), the expectation is that they should have only positive β_1_ values. For UIDS, assuming that they consist of an unknown mix of pyramidal cells and inhibitory interneurons, the expectation is that coefficients would be both negative and positive. However, all three populations show mostly positive coefficients and some negative ones as well (Fig. 13*A*). There was no significant difference between the different cell groups and *R*^2^ values (ANOVA, *F* = 0.07, *P* > 0.5) or absolute value of the rate coefficients (ANOVA, *F* = 0.67, *P* > 0.5). The *R*^2^ was significantly higher for cells with positive coefficients compared with negative coefficients (0.23 vs. 0.14; unpaired *t*-test, *P* < 0.001).

We found that 53% of all cells showed a significant correlation in their perimovement firing with movement onset time. This is likely to be an underestimate due to two reasons. First, there was a limited number of trials, so cells with a weak *R*^2^ would only come out as significant if they had a sufficiently large number of trials. This is confirmed in Fig. 13*B*, which plots the number of trials vs. *R*^2^. For cells with few available trials, the minimum detectable *R*^2^ was higher. A further reason is that some cells might be more engaged with the gripping part of the task, and as we were most interested in reach onset rather than squeeze, the period over which we measured rate was chosen to specifically minimize the influence of responses to squeeze. If grip onset was included as another variable in the model, the fraction of cells that were significantly (*P* < 0.02) correlated with reach onset and/or grip onset increased to 67%.

**Fig. 14.**
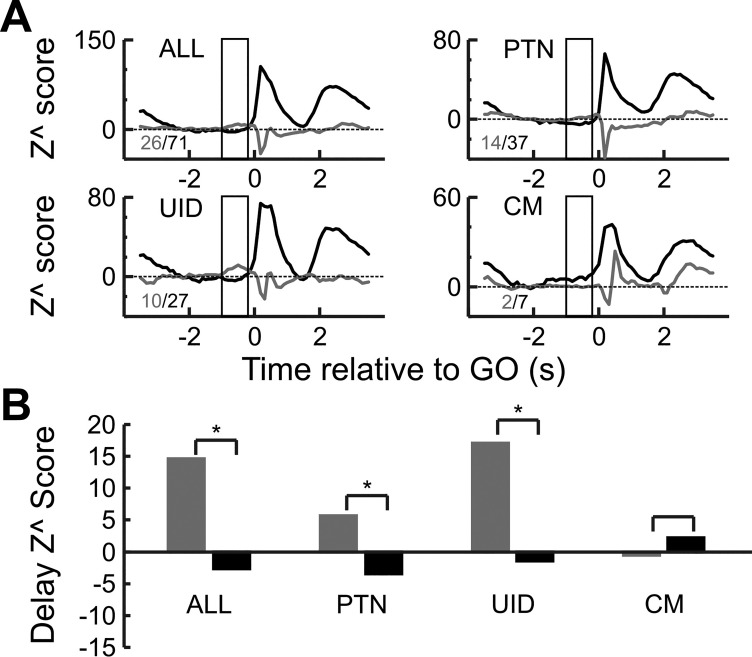
Relationship between rate coefficient and delay suppression. Only cells with significant correlation (*P* < 0.05) with movement onset are used. *A*: zPETH of all cells with positive correlation (black) and negative correlation (gray) relative to GO cue. Numbers within each subplot indicate the number of cells with each category. Box highlights the epoch (−1 to −0.2 s relative to GO cue) used to estimate the values in *B*. *B*: population *Z*^ scores for the different cell types during the instructed delay period for cells with positive (black) and negative (gray) correlation coefficient. *P value < 0.005 (unpaired *t*-test), comparing positive with negative cells.

Figure 14 shows the population data as mean zPETHs for cells with positive and negative β_1_ values. These results only consider cells with a significant correlation with movement onset. Figure 14*A* shows the population zPETH of cells with positive and negative β_1_ values. Cells with positive coefficients tended to show suppressed neural firing during the delay period (*n* = 71, epoch *Z*^ score: −2.5) compared with cells with negative coefficients (*n* = 26, epoch *Z*^ score: 14.6), and this difference was significant (*P* < 0.00001, unpaired *t*-test). A similar pattern was seen for PTNs (β_1_ > 0, *n* = 37, epoch *Z*^ score: −3.4 and β_1_ < 0, *n* = 14, epoch *Z*^ score: 5.7) and UIDs (β_1_ > 0, *n* = 27, epoch *Z*^ score: −1.4 and β_1_ < 0, *n* = 10, epoch *Z*^ score: 17.1). For both cell groups these differences were significant (both *P* values < 0.005, unpaired *t*-tests). CM cells showed the inverse pattern (β_1_ > 0, *n* = 7, epoch *Z*^ score: 2.2 and β_1_ < 0, *n* = 2, epoch *Z*^ score: −0.4), but the difference was not significant (*P* value > 0.4, unpaired *t*-test), most likely because of only two cells showing a negative β_1_. Figure 14*B* shows the population *Z*^ scores for the instructed delay period for the different cell populations.

To summarize, when neurons are characterized as “positive” or “negative” based on their correlation with movement onset, a substantial minority of PTNs violated the expectations of the M1 gating model for excitatory cells, in that they showed a negative relationship of their activity with movement onset. Their firing profile (rate suppression during movement and maintained rate during the delay) was instead what would be expected from cells involved in suppressing movement.

## DISCUSSION

The results of this study show that during an instructed delay task M1 corticospinal firing is suppressed during the delay, but this suppression is not distributed evenly across different neural populations. Furthermore, a substantial fraction of PTNs were suppressed during movement instead of during the delay period, and this is a profile that might be expected from inhibitory cells, not from excitatory pyramidal neurons. There was also a significant relationship between the upcoming reaction time and the depth of the rate suppression during the delay period—the less the rate suppression seen in PTNs, the faster the reaction time.

### Corticospinal Suppression During Action Preparation

Motor inhibition has been shown to impact on several facets of motor control including action selection, action preparation, as well as action stopping. The present study is most relevant to the role of reduced corticospinal excitability during action preparation, as the task used here was an instructed delay paradigm that requires the animal to withhold an action until a GO cue. In that respect, the main finding of corticospinal suppression during a preparatory delay is in line with the large body of literature showing similar results in humans (Duque et al. 2017).

**Fig. 15.**
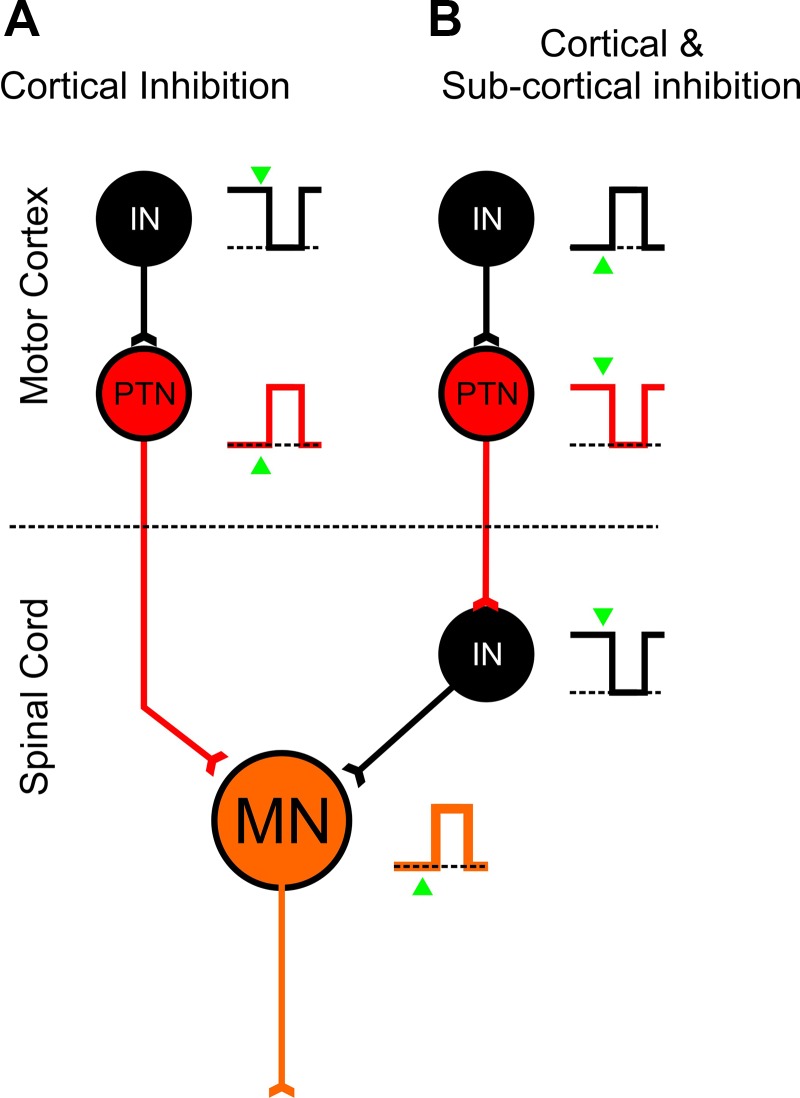
Cortical and subcortical inhibitory gating models: schematic for “expected” responses of inhibitory interneurons (black), PTNs (red), and motoneurons (orange) during delay and during movement. Dashed lines indicate baseline level of activity, and triangles indicate the GO cue. *A*: simple cortical inhibitory gate model, where inhibition is mainly driven by cortical inhibitory cells. *B*: prediction when subcortical inhibition (for example at the level of the spinal cord) is also included—note that in this case the expected rate responses for PTNs and interneurons are now the reverse of those predicted by *A*. Note that these are not mutually exclusive processes and both could occur together.

Most models for motor inhibition have the corticospinal system at their core as driving muscles and movement (Fig. 15*A*). This is not an unreasonable assumption, as corticospinal cells are exclusively excitatory and have direct and potent connections onto motoneurons (Fetz and Cheney 1980; Lawrence et al. 1985; Lawrence and Hopkins 1976; Porter 1985). The model of corticospinal suppression during action preparation would then be that the corticospinal system is active during movements but less active or suppressed during preparation and that cortical inhibitory interneurons would show an inverse pattern, i.e., suppression during movement and maintained rate during preparation. A caveat in this assumption, though, is that the corticospinal system does not just contact motoneurons but also contacts several other spinal neurons and circuits, many of which are inhibitory (Alstermark et al. 1984; Illert and Tanaka 1978; Isa et al. 2007; Jackson et al. 2006; Jankowska and Tanaka 1974; Nicolas et al. 2001; Rudomin and Schmidt 1999; Wu and Perlmutter 2013). If we now include this in the model (Fig. 15*B*), we can see that we might also expect to find that there is evidence for corticospinal suppression during movement and, contrastingly, facilitation of inhibitory interneurons during movement. This suggests that even within the “gating” model we cannot safely make predictions about the expected rate profile of interneurons and pyramidal cells. By looking at how cell activity around the time of movement onset was correlated with behavior, most PTNs fit a “positive” profile (Fig. 15*A*), but there were some that instead fit a profile that suggested that they were driving inhibitory circuits downstream (Fig. 15*B*). If this is true, then this suppression is likely to be mediated downstream of the motor cortex, such as the brain stem (Du Beau et al. 2012) and spinal cord (Harel et al. 2008; Prut and Fetz 1999; Shalit et al. 2012; Zinger et al. 2013).

The schematic shown in Fig. 15 is, of course, an extreme oversimplification and does not even take into account the fact that the same PTNs can contact both excitatory and inhibitory elements within the spinal cord (Cheney et al. 1982, 1985; Nishimura et al. 2013). It does, however, offer a starting point in explaining the observation that there are PTNs whose firing patterns are best explained if they were most interested in movement suppression—movement onset does not occur until these cells reduce their firing rate.

This study has focused on the cortex, but there is also a wealth of evidence for subcortical areas contributing to action preparation and motor inhibition such as the basal ganglia (Aron et al. 2007), brain stem, as well as the spinal cord. The brain stem reticular formation, unlike the corticospinal system, has inhibitory (glycinergic) reticulospinal cells and not just excitatory ones (Du Beau et al. 2012). This would allow a direct route of inhibition, above and beyond reticulospinal actions on spinal inhibitory interneurons (Engberg et al. 1968; González et al. 1993; Jankowska et al. 1968; Quevedo et al. 1995; Rudomín et al. 1983; Rudomin et al. 1986). Whether this dedicated inhibitory brain stem system receives cortical inputs is not known (Magoun 1950; Rhines and Magoun 1946), but many M1 corticospinal cells send collaterals to the reticular formation in addition to dedicated corticoreticular projections (Kably and Drew 1998; Matsuyama et al. 2004; Matsuyama and Drew 1997). As such, all the elements are there to allow the reticular formation to be engaged in motor inhibition, and the diverse projection of many reticulospinal cells (Kakei et al. 1994; Matsuyama et al. 1999; Mitani et al. 1988; Peterson 1979) would seem well suited for a broad impact across many motor pools.

Recordings from spinal interneurons in behaving monkeys have highlighted that preparatory activity also occurs at the level of the spinal cord, and this may be related to multiple delay processes (Prut and Fetz 1999) occurring at the spinal level. Furthermore, studies have also suggested that the spinal cord is likely to make unique contributions to delay processes, as spinal activity is not simply a relay of descending corticospinal commands (Shalit et al. 2012). In human studies, indirect evidence for spinal delay inhibition can be seen by transient suppression of the H reflex toward the end of the delay period (Duque et al. 2010; Hasbroucq et al. 1999; Touge et al. 1998). This fits with the finding that some corticospinal cells showed delay and movement responses that could be explained if they were contacting downstream inhibitory elements.

### Corticomotoneuronal Facilitation During Action Preparation

The suppression seen in PTNs was not universal—as a group, CM cells showed consistent facilitation during the delay period, which is in contrast to what the rest of the recorded PTNs showed (Figs. 4 and 5). This could perhaps be due to anticipatory muscle activity that we failed to detect, or in muscles we did not record from, but as the CM cells were recorded within the same sessions as PTNs (Fig. 7) this would not be enough to explain the difference between the two groups of cells.

The facilitation in CM cells might seem to contradict the response suppression to TMS seen in humans. However, TMS activates the corticospinal system mostly indirectly for the coil orientations used in most studies (Di Lazzaro et al. 2004; Edgley et al. 1997). As such, it would still be possible to have a net suppression of a facilitated CM system, if the presynaptic (to CM cells) elements that are activated by TMS are themselves suppressed. In addition, increased firing rate alone does not determine excitability—during a similar precision grip task in monkeys (Baker et al. 1995), the corticospinal system was most responsive to TMS during the hold period and not during dynamic movement, which is when PTNs and CM cells tend to be most active. Furthermore, although it is likely that muscle responses to TMS are mediated through the CM system, it has been shown recently that there is also likely to be a reticulospinal contribution (Fisher et al. 2012) and a spinal contribution as well (Bunday et al. 2014; Cirillo and Perez 2015) and that this is sensitive to the state of spinal inhibitory circuits. Whether this is the case for the suppression of responses to TMS seen during action preparation has not yet been shown.

The functional role of the CM facilitation is unclear, but one possibility is that it represents a process of action preparation occurring concurrently with action suppression (carried out by non-CM PTN cells). The delay between a stimulus and a response even in simple reaction time tasks is far too long than that expected from conduction delays and synaptic relays (Carpenter 1999; Thompson et al. 1996), showing that it takes time for cortical machinery to bring about action. Speculatively, in an instructed delay task such as the one used here, both action preparation and action suppression could occur concurrently if mediated through partly different neural systems. If the processes occurred serially, it would likely take added time for cortical circuits to shift from a suppressed state to facilitation. We found that for trials with a faster reaction time there was a significantly larger response in CM cells, combined with a significantly reduced suppression in other PTNs (Fig. 8). The combination of suppression of the non-CM PTNs with the facilitation of the CM cells thus might represent multiple preparatory processes hypothesized to occur during impulse control in the cortex (Bestmann and Duque 2016) and spinal cord (Prut and Fetz 1999).

### Corticospinal Suppression During Movement Execution

The finding of suppression of corticospinal activity during movement has been reported by others in the field (Evarts 1968; Evarts and Tanji 1976; Kraskov et al. 2009, 2014; Quallo et al. 2012; Vigneswaran et al. 2013). In one of the early studies (Evarts 1968) on corticospinal activity during movement, Evarts reported PTNs that suppressed their activity during a voluntary wrist movement. As the task used in that study consisted of wrist flexion and extension movements, suppression in PTN firing was associated with being involved in reducing the drive to the muscles antagonistic to the planned movement. This is less likely to be the case in the present task, as all the muscles tended to be coactivated at the time of reach (Fig. 2, *A* and *B*) from rest. While we did not record from all muscles controlling the upper limb and so cannot exclude that there was a postural muscle that was active during the delay period and silent during movement, the lack of EMG during the delay period suggests against this possibility. As the homepads did not require any active force in order to be pressed (the weight of the animal’s hand was sufficient), if any force was applied this would likely have been picked up by the EMGs that we already recorded from.

Suppression of PTN activity has also been reported in monkeys performing fine manipulative tasks either with the fingers or during tool use (Quallo et al. 2012). The suppression of PTNs during naturalistic movements could represent pruning of unwanted activation of certain muscles, or it could correspond to suppression of EMG activity. During naturalistic movements there is often a complex temporal pattern of muscle activity, which includes EMG suppression (Quallo et al. 2012). Suppression of PTN activity is also very prominent in the mirror neuron system (Kraskov et al. 2014) in both M1 (Vigneswaran et al. 2013) and premotor cortex (Kraskov et al. 2009), where it has been suggested that it may serve a role in suppressing unwanted movements during action observation.

Our results would agree with these previous findings, and extend them to a more general role of the corticospinal system in movement suppression, as speculated previously (Kraskov et al. 2009). Considering the schematic model shown in Fig. 15, the corticospinal system could contribute to withholding action in two ways. For PTNs whose activity results in an increase in the excitability of motoneurons (either directly or indirectly through other excitatory pathways; Fig. 15*A*), these cells would need to be suppressed during periods when movements need to be prevented—in this task this would be during the delay period and these would correspond to the “positive cells” identified by the regression and movement index analysis (Figs. 10 and 14). If PTNs were involved in driving inhibition at the subcortical or spinal level as mentioned previously, we would instead expect that they should be most active during periods when movement needs to be prevented and least active when movement needs to occur. This is exactly the pattern we observe in “negative cells,” whereby they show increased firing during the delay period and suppressed firing during the movement, and this firing is negatively correlated with behavior (Figs. 10 and 14).

This raises the question of whether a specific group of PTNs form a “generic” system for voluntary movement suppression (Ghosh et al. 2014; Greenhouse et al. 2015b) or if this falls to a different group of cells for different movements (Duque et al. 2010). We cannot address that here, but, interestingly, when the same CM cells were studied for two different tasks (Quallo et al. 2012) suppression was not uniform across tasks—CM cells that were suppressed during tool use were not necessarily suppressed during precision grip. However, the rake task used in that study showed suppression in some muscles (Figure 7 of Quallo et al. 2012) during the movement, which might explain the higher fraction of CM cells showing reduced firing during that task.

### Comparison with Previous Work on Gating Within Primate M1

The results of this study are different from others that have failed to find evidence of any gating within M1 (Kaufman et al. 2013). There are many possible reasons why that might be the case. One possibility is that the nature of the task used here was very different from that used in those studies. The task used here was much more similar to instructed delay tasks used in human studies, where there was a long cue and delay period in which the animal had to hold still, unlike the “maze” task, for which the instructed delay varied from 0 to 1 s. In humans, the duration of the delay period has an impact on the amount of motor suppression observed during preparation (Lebon et al. 2016), and indeed in reaction time tasks with no delays there is instead evidence for facilitation in M1 just before movement onset (Davey et al. 1998; Leocani et al. 2000). Suppression during the delay period might have been more pronounced in Kaufman et al. (2013) if only trials with longer delays were used for the analysis.

A further task difference is that in our case the movement was ballistic and was always to the same target and successful performance most likely relied heavily on somatosensory feedback. The “maze” task was far more complex, as upcoming movements were not always to the same target and successful performance relied, in addition to peripheral somatosensory feedback, on the continuous use of visual feedback as well. There is ample evidence to show that M1 excitability can be very sensitive to the visuomotor demands of the task (Pruszynski 2014; Pruszynski et al. 2008; Pruszynski and Scott 2012) and that motor inhibition does show some dependence on task complexity (Greenhouse et al. 2015a), being reduced for more complex movements.

Finally, cells in this study were segregated on the basis of their anatomical differences, and this revealed that suppression during the delay period is not equally distributed across all cell types. Even within the corticospinal system there is a substantial difference between subpopulations—many non-CM PTNs showed robust suppression, while most CM cells showed robust facilitation during the delay period. When all cells are combined together, the evidence for suppression at the population level is much weaker (Fig. 4). Although previous work attempted to identify interneurons by spike width, this can be problematic in motor areas, as fast corticospinal cells show spike widths comparable to those shown by inhibitory interneurons (Vigneswaran et al. 2011). UIDs, which were most likely made up of a mix of pyramidal cells from various subpopulations, showed the most inconsistent evidence for suppression.

### Final Conclusions

The results shown here provide some support for “gating” in M1, but this on its own does not disprove “dynamical gating,” as the mechanisms could coexist (Kaufman et al. 2014). Concurrent facilitation and suppression, even of different cell types within M1, could still be interpreted as the neural variability predicted by operation within “output null” regimes (Kaufman et al. 2013, 2014). But this does raise the issue of how different neuronal populations fit within the dynamical framework.

There are a multitude of reasons why it makes sense to view the brain as a dynamic system, but one of the biggest strengths of this approach is that instead of trying to match the neural data to whatever variables the experimenter thinks are important, it allows the experimenter to find whatever hidden variables best explain the data and then see how these are related to the experimenter’s variables of interest. However, this dynamical system is still implemented by a neural architecture made up of several distinct neuronal elements. We could assume that their identity does not matter, but given the huge investment during development to make sure cells are in the right place and connect to the appropriate area, this seems unlikely. There are several examples of specific pyramidal subpopulations of cells showing distinct connectivity and firing patterns (Harris and Shepherd 2015). For example, callosal neurons have been shown in a variety of species to have very low basal firing rates, in contrast to other pyramidal cells (Beloozerova et al. 2003a, 2003b; Soteropoulos and Baker 2007; Swadlow 1994), and this does constrain their potential roles during movement (Soteropoulos and Baker 2007). Finding that CM cells and non-CM PTNs behave differently during a preparatory delay further reinforces this consideration. The ensuing challenge for deciphering how preparatory processes operate before movement is to determine how distinct neural elements fit within the dynamical motor system.

## GRANTS

The author was funded by a Medical Research Council New Investigator Award.

## DISCLOSURES

No conflicts of interest, financial or otherwise, are declared by the author.

## AUTHOR CONTRIBUTIONS

D.S.S. conceived and designed research, performed experiments, analyzed data, interpreted results of experiments, prepared figures, drafted manuscript, edited and revised manuscript, and approved final version of manuscript.
